# Response shift in results of patient-reported outcome measures: a commentary to The Response Shift—in Sync Working Group initiative

**DOI:** 10.1007/s11136-020-02747-4

**Published:** 2021-01-22

**Authors:** Mirjam A. G. Sprangers, Tolulope Sajobi, Antoine Vanier, Nancy E. Mayo, Richard Sawatzky, Lisa M. Lix, Frans J. Oort, Véronique Sébille, The Response Shift—in Sync Working Group

**Affiliations:** 1 Department of Medical Psychology, Research Institute Amsterdam Public Health, Amsterdam University Medical Centers, Location AMC, Meibergdreef 15, J3-211, 1105 AZ Amsterdam, The Netherlands; 2 Department of Community Health Sciences, University of Calgary, Calgary, Canada; 3 Inserm—University of Nantes—University of Tours, UMR 1246 Sphere “Methods in Patient-Centered Outcomes and Health Research”, Nantes, France; 4 Center for Outcomes Research and Evaluation, McGill University, Montreal, Canada; 5 Division of Clinical Epidemiology, Department of Medicine, McGill University Health Centre Research Institute, Montreal, Canada; 6 School of Nursing, Trinity Western University, Langley, BC Canada; 7 Centre for Health Evaluation and Outcome Sciences, University of British Columbia, Vancouver, BC Canada; 8 Department of Community Health Sciences, University of Manitoba, Winnipeg, Canada; 9 Research Institute of Child Development and Education, University of Amsterdam, Amsterdam, The Netherlands; 10 UMR INSERM 1246, SPHERE “methodS in Patient-Centered Outcomes and HEalth ResEarch”, University of Nantes, University of Tours, Nantes, France

**Keywords:** Response shift, Definition, Theory, Method, Meta-analysis, Decision-making

## Abstract

**Purpose:**

The Working Group undertook a critical, comprehensive synthesis of the response shift work to date. We aimed to (1) describe the rationale for this initiative; (2) outline how the Working Group operated; (3) summarize the papers that comprise this initiative; and (4) discuss the way forward.

**Methods:**

Four interdisciplinary teams, consisting of response shift experts, external experts, and new investigators, prepared papers on (1) definitions and theoretical underpinnings, (2) operationalizations and response shift methods, (3) implications for healthcare decision-making, and (4) on the published magnitudes of response shift effects. Draft documents were discussed during a two-day meeting. Papers were reviewed by all members.

**Results:**

Vanier and colleagues revised the formal definition and theory of response shift, and applied these in an amended, explanatory model of response shift. Sébille and colleagues conducted a critical examination of eleven response shift methods and concluded that for each method extra steps are required to make the response shift interpretation plausible. Sawatzky and colleagues created a framework for considering the impact of response shift on healthcare decision-making at the level of the individual patient (micro), the organization (meso), and policy (macro). Sajobi and colleagues are conducting a meta-analysis of published response shift effects. Preliminary findings indicate that the mean effect sizes are often small and variable across studies that measure different outcomes and use different methods.

**Conclusion:**

Future response shift research will benefit from collaboration among diverse people, formulating alternative hypotheses of response shift, and conducting the most conclusive studies aimed at testing these (falsification).

## Introduction: synthesis, please

Three decades ago, Breetvelt and Van Dam [1] noticed the recurrent finding that patients with cancer or other diseases did not report a lower level of quality of life (QoL) and happiness, or more anxiety and depression than healthy individuals, despite physical malaise, and contrary to the expectations of their healthcare providers. They were the first to link this ‘underreporting’ of malady to response shift, a term coined by Howard et al. [2]. They posited that as a consequence of adaptation to their disease, patients may change their internalized standard with which they rate their well-being, rendering the response scales over time and between healthy groups and patients potentially incompatible and invalid. They warned their readers that “Until an empirically proven solution to this problem has been found, we recommend that answers in questionnaires concerned with quality of life, psychological distress and the like should be approached with due caution” [1], p. 981.

The challenge to search for solutions in the field of patient-reported outcomes (PROs) was accepted in the form of a series of papers on response shift in Social Science & Medicine in 1999 [3], that was also part of a first tome on response shift, published in 2000 [4]. Based on the definitions provided by Howard and colleagues [2] in the area of educational interventions and Golembiewski et al. [5] in the field of organizational change, Sprangers and Schwartz [6] proposed that response shift refers to a change in the meaning of one's self-evaluation of a target construct as a result of: (a) a change in the respondent's internal standards (recalibration); (b) a change in the respondent's values (reprioritization); or (c) a redefinition of the target construct (reconceptualization). Armed with this working definition of response shift, a preliminary theoretical model [6], a range of promising assessment approaches for detecting response shift [7], and some examples of applied implications [8–10], research on response shift was equipped to start and grow. And so it did. The last two decades witnessed an expanding interest in response shift with proposals for refined definitions and theoretical models [11–14], a proliferation of new response shift detection methods [15], and a wealth of empirical studies in a range of clinical populations [15]. Since the early years of this millennium, response shift is a recurrent topic at the annual conferences of the International Society for Quality of Life Research (ISOQOL) in the form of workshops, symposia, oral and poster presentations and the subject of a Special Interest Group. Empirical studies and reflections on response shift were published in special series in 2009 in the Journal of Clinical Epidemiology [16], and in 2011 and 2019 in Quality of Life Research [12, 17]. Response shift is currently taught by many response shift experts in various curricula at their home institutes and is a term that meets recognition and interest in clinical audiences.

Response shift did not remain uncriticized and controversies were also appearing in the literature, which we took as an indication that the field was maturing. For example, Ubel, Peeters and Smith [18] argued that research into response shift had been hampered by conceptual confusion since the term ‘response shift’ is an amalgam of different sources of measurement error. They went so far as to propose abandoning the term response shift altogether. Their article prompted three passionate responses that provided arguments for retaining this label [19–21]. However, Ubel and colleagues did have a point by referring to the conceptual confusion. Earlier work already noticed that people attach different meanings to the term response shift. At that time, Frans Oort distinguished six dimensions of cross-talk that are relevant to date, where response shift could be considered as bias versus meaningful change, measurement versus subject characteristics, explanation of counter-intuitive findings versus a phenomenon itself, temporary change versus long-lasting or permanent change, a result of an event versus the mere passage of time, and ‘something’ unrelated to health versus ‘something’ exclusively related to health [14, 22]. Such diversity might be unavoidable and even helpful in the early, exploratory phases of response shift research. However, allowing the same term to encompass these different ideas will not only lead to continuous miscommunication and confusion but ultimately hinder progress.

It was therefore deemed high time for a critical, comprehensive appraisal and synthesis of the work to date on four interrelated topics. First, given the publication of several definitions of response shift as well as a range of theoretical reflections and conceptual models on response shift aimed at explaining the phenomenon [6, 11, 14], there is a need for a synthesis of these definitions and theories with the goal to identify common ground and differences and to propose amendments if deemed needed. Second, over the years, several response shift methods have been developed [15, 23] that aim to detect response shift, but differ in their operationalization of response shift (i.e., how response shift is evidenced in the results). Bringing previous descriptive reviews [15, 23] a step further, a critical analysis is needed on the operational definitions and assumptions underlying these response shift methods and the alternative explanations of the results. Third, an important goal of using patient-reported measures (PROMs) in healthcare is to inform decision-making. Since response shift can affect PROM results, it can indirectly influence such decision-making. However, the implications of response shift for healthcare decision-making have rarely been explored [24]. A theoretical analysis is needed of potential implications of response shift for a wide range of healthcare decisions. Finally, the only meta-analysis was conducted on papers published up to 2005, which was necessarily limited to the then-test approach as that was the most widely used method at that time [25]. Since then statistical methods have been developed and used that allow for computation of effect sizes [15], warranting a new meta-analysis.

The overall objective of the current initiative was to critically examine response shift research in outcomes of PROMs, including QoL. We thereby focused on: (1) the definitions and theoretical underpinnings of response shift (what is it?), (2) the operationalizations and response shift detection methods, response shift methods for short (how do you measure it?), (3) implications of response shift for healthcare decision-making based on PROMs (how does it affect decision-making?); and (4) the magnitude of the response shift effects found (what is its empirical evidence?). For all four areas three key questions were asked: What do we know? Can different views be synthesized? What are the gaps in our knowledge that require future research?

## The Response Shift—in Sync Working Group

To pay credit to the diversity of conceptual and operational definitions and suggested response shift methods, we believed that such a synthesis would require the effort of a wide range of response shift experts to bring a range of different disciplinary and clinical perspectives to the topic. First and foremost, we needed their combined expertise. There was also another important reason. In their rejoinder Ubel and colleagues [26] wrote: “We expect that the ideas we present here will be controversial. Some scholars have made reputations for themselves by disseminating the concept of response shift” (p. 470). This sentence stung. Probably because it was true. Most of us have worked for a long time in the area of response shift, have provided definitions, contributed to theory, and/or developed response shift methods and conducted empirical research. This may have made us attached and therefore biased toward our own ideas. This all too human attitude was described by Chamberlin, a geologist, at the end of the 19th century. He wrote: “The moment one has offered an original explanation for a phenomenon which seems satisfactory, that moment affection for his intellectual child springs into existence, and as the explanation grows into a definite theory his parental affections cluster about his offspring and it grows more and more dear to him ….. There springs up also unwittingly a pressing of the theory to make it fit the facts and a pressing of the facts to make them fit the theory ….” (quote cited in [27], p. 350). We felt that collaboration among different experts would keep the respective preconceived ideas in control and diminish the chance that one viewpoint would unjustifiably dominate. We hoped that by sharing and discussing each and every insight, the ideas would be increasingly seen as shared rather than individual intellectual property. However, bringing together excellent and passionate researchers on response shift required additional measures to counteract possible vested interests and preconceived ideas. We also needed external experts, including seasoned scholars, clinicians, and patient research partners who had not been involved in response shift research to obtain independent and diverse views. Moreover, inclusion of young investigators new to response shift research was expected to generate naive, and therefore important, questions. It was also a means to stimulate a new generation of researchers.

Following these starting points, we brought together an international and interdisciplinary team of researchers across a wide range of institutions, called the Response Shift—in Sync Working Group (see Appendix). The number of participants was limited to 26 to keep the size of the group manageable and enabling meaningful and focused discussions. The choice of the participants was based on expertise and reputation. Eleven out of 26 team members of the Working Group are from Canada, which is, in part, due to the fact that senior researchers introduced their PhD students and post-docs. Moreover, almost all response shift experts are linked directly or indirectly to ISOQOL. It should be noted that the group is not speaking for any particular entity, only for themselves. The work described here presents the collaborative effort of these researchers, paying credit to the extant work on response shift as best as they could.

The Working Group participants were combined to form four interdisciplinary teams related to the four identified response shift topics. The composition of the teams was informed by the desire to spread expertise and reduce possible dominance of ideas. If members had jointly co-authored response shift papers previously, they were assigned to different groups wherever possible.

Each team had a designated chair, at least two response shift experts, at least one independent external expert, and at least one new investigator (see Table 1 for team compositions). The teams differed in magnitude. The largest team was assigned to the third paper on the implications of response shift for healthcare decision-making. Given the novelty of this topic we needed more external experts, including a health economist, a patient-centered outcomes researcher, a patient research partner, a research consulting representative, and clinicians in addition to methodologists. Whereas we intended to make the teams mutually exclusive, in the end some response shift experts were part of both Team 2 on response shift methods and Team 4 on meta-analysis, given the connection between these topics. Finally, in the course of the writing process some team members also contributed substantially to other papers warranting co-authorship, leading to more overlap among the teams.

**Table 1:** Response shift in Sync—Working Group participants grouped according to member category and original assignment to writing team

Writing teams	Team 1—Formal definition and revised model	Team 2—Operationalization and response shift methods	Team 3—Implications for healthcare decision-making	Team 4—Systematic review and meta-analysis of effect sizes
Team chairs	Nancy Mayo, CA	Véronique Sébille, FR	Rick Sawatzky, CA	Tolulope Sajobi, CA
Independent, external experts	Jan R. Böhnke, DE, UK	Cecile Janssens, NL, US	Cynthia Chauhan, US Lori Frank, US Wilbert van den Hout, NL	Olu Awosoga, CA
Response shift experts	Leah McClimans, US Frans Oort, NL Antoine Vanier, FR	Lisa Lix, CA Tolulope Sajobi, CA Rick Sawatzky, CA Mathilde Verdam, NL	Ruth Barclay, CA Sandra Nolte, DE, UK Mirjam Sprangers, NL	Amélie Anota, FR Lisa Lix, CA Rick Sawatzky, CA
New investigators	Bernice Gulek, US Nikki OW, CA	Olawale Ayilara, CA	Lene Kongsgaard Nielsen, DK Jae-Yung Kwon, CA	Anita Brobbey, CA Oluwaseyi Lawal, CA

*CA* Canada, *DE* Germany, *DK* Denmark, *FR* France, *NL* Netherlands, *UK* United Kingdom, *US* United States

The teams were asked to prepare draft summary documents, which were circulated among the entire working group prior to a 2-day meeting that was held in September 26–28, 2019 in Castle Oud Poelgeest in the Netherlands. The meeting format was targeted to promote the discussion and refinement of the four draft documents. Each team gave a 30-min presentation and had a 90-min open discussion in plenary. Hence, each topic received 120 min of the group's collective attention. At the end of the second day, two hours were reserved for team discussions to synthesize the ideas forwarded and plan the subsequent writing phase. The final one-hour slot at the end of the meeting was a plenary session devoted to wrapping up the discussions, planning the preparation of the papers and assigning tasks to the participants.

To manage the working group, a core group was composed of the two co-chairs, MS and VS, the team chairs and first authors if these roles were separated, TS, NM, AV, and RS, and two additional experienced experts in this field, LL and FO. The overall organization of the working group was led by the co-chairs, who were supported by the core group with whom they had regular conference calls. The co-chairs had additionally regular bilateral contact with the team leaders/first authors about their respective papers. At critical phases, draft versions of the documents were reviewed by the co-chairs to ensure focus, avoid overlap, and safeguard timing. The semi-final papers were circulated among the full working group to seek their input before submission. The papers are therefore the result of the collaborative effort of the Response Shift – in Sync Working Group at large.

## Overview of papers

### Team 1: formal definition and revised model

Vanier and colleagues [28] took up the challenging task to disentangle the definitional confusion around response shift and sorting out the various theoretical viewpoints. They first made an overview of the extant definitions and theories of response shift. To further solve the definitional tangle, they also started to make an inventory of concepts that are related to, but distinct from response shift and that are sometimes confused with response shift. They also explicated how these concepts relate to response shift. This inventory may not be exhaustive and can be expanded or fine-tuned with time.

They subsequently identified three major predicaments in the response shift definitions and theories. First, the formal definition of response shift proposed by Oort [13] and Oort and colleagues [14] was formulated as a violation of conditional independence. However, this conceptualization may be too general because it also encompasses other explanations underlying this violation. Moreover, its complex, statistical basis may have prevented wide adoption. Vanier and colleagues therefore further specified and clarified this formal response shift definition and proposed alternative wording that is easier to understand. Second, although response shift is a time-dependent phenomenon related to change, the extant models only visualize one time point. Third, extant models do not distinguish the target construct (e.g., QoL) from its measure (e.g., PROM) despite response shift targeting their relationship. Vanier and colleagues proposed a revised model in an attempt to solve these two latter predicaments. They created a model for a case in which PROs are measured at two points in time, distinguishing the measure from the construct under investigation. This model illustrates possible chains of causality explaining the level of the PROM and the construct at both times. They meticulously explicated the epistemic, methodological and practical assumptions underlying this model as the minimal conditions for the model to hold. The authors demonstrated that the model refers to real life experiences, by providing quotes from people describing these experiences in their own words as presented in the literature. They finally discuss the assumptions and implications of their revised definition and model for research on response shift and conclude that the proposed model lends itself for analytical and empirical examination, including refutation, given its explicit list of assumptions and hypothesized relationships.

### Team 2: operationalization and response shift methods

Based on previous reviews [15, 23], Sébille and her colleagues [29] identified 11 methods, including the then-test and appraisal method, representing the design approaches [7, 11], semi-structured interview exemplifying the qualitative approach [30], adaptation of the Schedule for the Evaluation of Individual Quality of Life (SEIQoL) illustrating the individualized approach [31], and vignette studies typifying preference-based approaches [32]. The remaining methods rely on various statistical methods. Within the framework of latent variable models, methods include Structural Equation Modeling (SEM) [13], Item Response Theory [33] and Rasch Measurement Theory [34]. Other frameworks not necessarily requiring modeling of latent variables encompass Relative Importance Analysis [35], Classification and Regression Tree [36], Random Forest Regression [37], and Mixed Models and Growth Mixture Models [38]. They critically appraised these methods regarding their implied definitions, operationalizations, the type of response shift they can detect, whether they can adjust for and explain response shift, their underlying assumptions, and alternative explanations of results.

The detailed inventory made clear how the different methods reflect different definitions and operationalizations of response shift, explaining why different methods may lead to different results and conclusions about the occurrence of response shift. Moreover, the specific assumptions underlying each method was specified and it was shown how different alternative explanations may account for the inferred response shift effects obtained by different methods. Sébille and colleagues concluded, not surprisingly, that no method is optimal in all situations as they each have specific limitations that need to be considered. The key message was that response shift results should not be taken for granted, and extra steps are required to make the response shift interpretation plausible. The authors recommended training in the application of response shift methods and rigorous study designs that control for alternative explanations. They also called for new research directions and suggested new statistical approaches for handling inter-individual variation and multiple time points. Finally, they argued that simulation studies are needed to assess and compare the ability of different methods to detect response shift appropriately when it is (or is not) simulated under different conditions (e.g., varying sample sizes and magnitude of the response shift effect). Such studies would also allow for investigations about the extent to which a method is robust to alternative explanations.

### Team 3: implications for healthcare decision-making

Sawatzky and colleagues [39] created a framework for considering the different ways in which response shift may impact healthcare decision-making at the level of the individual patient (micro), the healthcare organization (meso), and healthcare policy (macro). Building on the perspective of measurement validity [40] as an interpretive process, the authors used a hermeneutic perspective that focuses on how individuals derive meaning from text [41], to gain insights into response shift implications at the three levels of healthcare decision-making.

At the micro-level, patients’ self-reports need to be interpreted via dialog with the clinician to facilitate assessments of change while taking the possible occurrence of response shift into account. Such consultations may pertain to decisions about the choice of treatment, goals of care, and the need for additional interventions and/or supportive services.

Such decisions are also informed by published study results. A particular challenge regarding the use of such aggregated patient-reported data is that response shift may have occurred but was not accounted for, possibly leading to incorrect inferences and hence ill-informed decisions. At the meso level, individual PROM data should therefore be inspected for response shift before aggregating these data for decision-making regarding quality improvement, performance monitoring, and accreditation. At the macro-level, it is important to consider the conceptualization of health to know whether response shift needs to be controlled for when PROMs are used to inform healthcare coverage, including provision and reimbursement of health services.

Sawatzky and colleagues asserted that there is a critical need for guidelines and knowledge translation to avoid potential misinterpretations of PROM data and resulting biases in decision-making. Their framework with guiding questions provides a means to stimulate strategies that address the potential impacts of response shift at micro-, meso-, and macro-levels.

### Team 4: systematic review and meta-analysis of effect sizes

A recent scoping review of the literature up to 2016 [15] revealed that more than 80% of the empirical investigations of response shift adopted either then-test and/or Latent Variable Modeling approaches to test for response shift in PROM data. Sajobi and colleagues have now furthered this review by conducting a systematic search on studies published up to June, 2019 enabling a wider scope of response shift methods, populations, and PROM domains.

The aim was to describe and quantify response shift effect sizes in PROM data and to investigate the factors that explain variations in these effect sizes. Preliminary results indicate that the median response shift effect sizes varied per method and PROM domain and were all of a small magnitude (effect sizes < 0.40). The major finding, however, was that the heterogeneity in the data was so large that it precluded straightforward combining of the results. They therefore decided to split the paper into two. The first is a descriptive, systematic, comprehensive review describing the distribution of and variation in response shift effect sizes and characteristics of the included and excluded studies. Subsequently, a meta-analysis paper will be prepared which will report the pooled data, the results of the multi-level meta-regression analyses to evaluate the impact of study characteristics and reporting quality on variations in reported effect sizes, and the potential influence of publication bias. These two papers are not part of the special issue but will be submitted at a later point in time.

The resulting knowledge about the magnitude and variation in response shift effect sizes and conditions under which response shift is or is not detected will inform the future design of longitudinal studies and guide the selection of PROMs for specific populations. For example, if the aim is to gain more insight into the types of change, larger sample sizes are required to adequately power studies of response shifts, given the current evidence of generally small effect sizes.

## Diversity, alternative hypotheses, and falsification

With the Response Shift – in Sync initiative, we re-learned three interrelated lessons that may advance response shift research: the need for diversity, alternative hypotheses, and falsification. With respect to the first lesson (“need for diversity”), we came to realize that the field of response shift research is characterized by compartmentalization, with several groups working relatively independently within their own theoretical and/or methodological domain. People holding ideas and use methods within those purviews do not have to fear being confronted with unpleasant questions, logical inquiry and threatening alternative interpretations. The price of this calm coexistence is that the advancement of response shift research is impeded, and intriguing and stimulating questions are not likely asked [42]. The Response Shift—in Sync Working Group is the first ‘pluralistic’ initiative that aims to synthesize and further the work on response shift in ‘playful competition’ [42]. In a ‘pluralistic competition game’ as many researchers as possible are engaged in varied negotiations in what theories and response shift methods really imply rather than leaving such discussion to the originators. The crucial rule is that a theory and a response shift method must exist independently of their designer’s opinion. Everyone has an equal right to identify strengths and weaknesses, formulate alternative hypotheses, and evaluate the theory and response shift method against logical analysis and empirical evidence. Only when theories and methods are scrutinized and used by many different researchers can their full potential and weaknesses be revealed [42, pp. 126–127]. Indeed, the discussions among the diverse participants of the Working Group were found to be much needed: in the beginning they were characterized by confusion and misunderstanding, gradually evolving to clarity, revealing where views diverged and needed confrontation, finally progressing into more comprehensive ideas encompassing the points and counterpoints raised.

The role of the independent experts cannot be overestimated. They played a pivotal role in the discussions puncturing the consensus response shift experts shared. This experiential finding has a strong empirical basis in social psychology. For example, in a series of experiments, Philips and colleagues [43] showed that diverse teams with newcomers performed better than homogenous teams, albeit at the cost of confidence in their own performance and in the effectiveness of their interactions. Contrary to what one might expect, performance gains did not result from newcomers voicing new ideas per se. Rather, members of diverse teams were more willing to change their initial opinion than were members of homogeneous teams.

The second lesson (“alternative hypotheses”) resonated philosophy of science lectures. Those taught us the importance of inductive inference or the scientific method, originating from Francis Bacon in the 16th century. It is the systematic application of a series of consecutive steps: elucidating the phenomena and concepts involved, forming an inductive hypothesis, formulating alternative hypotheses, carefully designing and performing crucial experiments to test those alternative hypotheses, and repeating the procedure for other or finer tuned hypotheses that may still remain. Whereas PRO researchers use scientific methods in their day-to-day activities, the ideal of inductive inference is so far away from our daily work as researchers in PRO research, one may wonder whether it applies to our field at all. However, we believe that the next move that will likely advance response shift research is to ask ourselves what alternative explanations may account for each theoretical notion and result obtained by any response shift method. Vanier and colleagues [28] used a model to show how response shift may be induced and in turn affect changes in PROs (target construct) and PROM results, listing the underlying assumptions inherent to this model. Some of these assumptions may be reformulated as alternative explanations. They also documented concepts that are related to, but are distinct from response shift to better clarify what response shift does not entail. Sébille and her team [29] are the first to systematically specify the alternative explanations of the results obtained by each response shift method. The list may not be exhaustive yet and may need to be expanded. The key questions subsequent to these initiatives include: are there possible situations where response shift occurs but is not defined nor detected and are other situations imaginable where response shift is defined and/or detected but does not occur in reality?

The third lesson (“falsification”) brought us back to the early years of response shift research. When we started investigating response shift, the message to the scientific community was provocative. If response shift was a viable phenomenon our basic design to assess change in PROM results—the common baseline follow-up design—may have serious flaws. A message that understandably did not meet a warmhearted reception by social scientists. Interestingly, we always encountered interest and support from our clinical colleagues who recognized the phenomenon during their patient consultations. As a result, we were diligently demonstrating, both theoretically and empirically, that response shift was not a mere figment of our imagination but a finding that could be inferred from data or was voiced directly by patients. We felt we were constantly shouting ‘It is there, watch out’. Perhaps we have roared for too long and became prone to confirmation bias [44]. This habit for seeking confirmation is not restricted to response shift research and is also noted in other areas of science. As the physician Platt previously put it: “We measure, we define, we compute, we analyze, but we do not exclude.” [27, p. 352]. Since science only advances by attempts to disproof, response shift research can only mature if we also look for falsification [45]. For example, the statistical response shift methods use goodness of fit—the data fit the expectations—as evidence of the occurrence of response shift. However, this is a necessary but not a sufficient condition and such conclusion would only be warranted if observations are possible that are inconsistent with response shift and alternative explanations can be eliminated [46, 47].

With the list of carefully selected possible alternative hypotheses, we need to take ample time to ponder which study designs would be able to test these and conduct the most informative and conclusive studies. In this stage, the key questions are for theory: “What study could disprove the hypothesis?” and for study results: “What hypothesis does the study disprove” [27]. Self-evidently, these studies need to be replicated, in turn. The results of such studies will possibly lead to refinements of the definition, alternative theoretical models and adaptations to the response shift methods after which the entire empirical cycle will start all over.

We want to notice that there is a more mundane and modest version of disproof and refutation. Meta-analyses may nuance a phenomenon’s strength, often showing smaller effect sizes than hypothesized. This type of refutation is more common in day-to-day PRO research. We therefore would recommend that response shift results are reported in ways that would allow for meta-analyses and that the meta-analysis of Schwartz and colleagues [25] and the one prepared by Sajobi and colleagues be followed up more frequently than once every 14 years.

## Curious scrutiny

We recognize that response shift could be defined in different ways and that there are numerous possible theoretical models. We do not intend the proposed definition and model by Vanier and colleagues [28] to be exclusionary or normative. Moreover, definitions and theories are in constant flux and need for ongoing scrutiny and revision. The current series of papers is therefore just a phase in the scientific discourse on response shift, hopefully a helpful one, in the further maturation of response shift research. An open mind encompassing all available approaches is needed. This pious stance is more difficult than we tend to admit. There is increasing evidence that more numerate individuals use their reasoning competence selectively to conform their interpretation of research data most consistent with their political convictions [48]. This may *a fortiori* hold for opinions and ideas that are dear to us and we are passionate about, such as certain theoretical or methodological approaches to response shift. Science curiosity was found to counteract such biased information processing [49] a hopeful and reassuring finding. Curiosity may be considered a *conditio sine qua non* for conducting genuine science. To provide an extreme counter example, scientific fraudsters may be excellent researchers in their ability to conduct the entire scientific cycle, but they do not only neglect the mores of scientific integrity, they also lack curiosity in how the phenomenon under study works in reality.

With these lessons in mind, we need curious researchers, willing to confront other ideas, and have their own beliefs be confronted. This will be challenging as it is unpleasant to be disagreed with and it needs courage to be a dissenter. New investigators, not hindered by preconceptions, may take on a more prominent role. For example, our theoretical notions and response shift methods could be submitted to inspection in university curricula. As part of philosophy of science courses, students may dissect the theoretical models and courses on methodology, statistics or psychometrics may ask students to check the response shift methods. With such pluralistic, curious scrutiny we expect response shift research to advance at a rapid rate.

## Appendix: Members of the Response Shift—in Sync Working Group

- Amelie Anota, Department of Human and Social Sciences INSERM UMR 1098, Cancer Care Center Léon Bérard, Lyon, France;
- Oluwagbohunmi Awosoga, Faculty of Health Sciences, University of Lethbridge, Lethbridge, Canada;
- Olawale F. Ayilara, Department of Community Health Sciences, University of Manitoba, Winnipeg, Canada;
- Ruth Barclay, Department of Physical Therapy, University of Manitoba, Winnipeg, Canada;
- Jan R. Böhnke, University of Dundee, School of Health Sciences, Dundee, United Kingdom;
- Anita Brobbey, Department of Community Health Sciences, University of Calgary, Calgary, Canada;
- Cynthia Chauhan, Patient Representative, Wichita, KS, USA;
- Lori Frank, Behavioral & Policy Sciences, RAND Corporation, Arlington, VA, USA;
- Bernice G. Gulek, University of Washington, Harborview Medical Center, Seattle, WA, USA and Washington State University, College of Nursing, Spokane, WA, USA;
- Wilbert van den Hout, Medical Decision Making, Department of Biomedical Data Sciences, Leiden University Medical Center, Leiden, The Netherlands;
- A. Cecile J. W. Janssens, Department of Epidemiology, Rollins School of Public Health, Emory University, Atlanta, GA, USA;
- Lene Kongsgaard Nielsen, Department of Haematology, Quality of Life Research Center, Odense University Hospital, Odense, Denmark and Department of Internal Medicine and Cardiology, Regional Hospital Viborg, Viborg, Denmark;
- Jae-Yung Kwon, School of Nursing, University of British Columbia, Vancouver, Canada;
- Oluwaseyi Lawal, Department of Community Health Sciences, University of Calgary, Calgary, Canada;
- Lisa M. Lix, Department of Community Health Sciences, University of Manitoba, Winnipeg, Canada;
- Nancy Mayo, Department of Medicine, Division of Clinical Epidemiology, Center for Outcomes Research and Evaluation (CORE), McGill University, Montreal, Canada;
- Leah McClimans, Department of Philosophy, University of South Carolina, Columbia, SC, USA;
- Sandra Nolte, ICON GmbH, Munich, Germany and Charité – Universitätsmedizin Berlin, corporate member of Freie Universität Berlin, Humboldt-Universität zu Berlin, and Berlin Institute of Health, Medical Department, Division of Psychosomatic Medicine, Berlin, Germany;
- Frans J. Oort, Research Institute of Child Development and Education, University of Amsterdam, Amsterdam, The Netherlands;
- Nikki Ow, School of Physical and Occupational Therapy, Center for Outcomes Research and Evaluation (CORE), McGill University Montreal, Canada;
- Tolulope T. Sajobi, Department of Community Health Sciences and O'Brien Institute for Public Health, University of Calgary, Calgary, Alberta, Canada;
- Richard Sawatzky, School of Nursing, Trinity Western University, Langley, Canada;
- Véronique Sébille, UMR INSERM 1246, SPHERE "methodS in patient-centered outcomes and HEalth ResEarch", University of Nantes, University of Tours, Nantes, France;
- Mirjam A. G. Sprangers, Department of Medical Psychology, Amsterdam University Medical Centers, Research Institute Amsterdam Public Health, Amsterdam, The Netherlands;
- Antoine Vanier, UMR INSERM 1246, SPHERE "methodS in patient-centered outcomes and HEalth ResEarch", University of Nantes, University of Tours, Nantes, France;
- Mathilde G. E. Verdam, Department of Methodology and Statistics, Institute of Psychology, Leiden University, Leiden, The Netherlands.
